# The Digital Entrepreneurship Era: How to Motivate Innovativeness in Middle Management Teams? The Vertical Organisational Pervasiveness of Chief Executive Officer Entrepreneurial Orientation

**DOI:** 10.3389/fpsyg.2022.775558

**Published:** 2022-03-30

**Authors:** Xu Zhang, Yueyue Liu, Xiulin Geng, Danxia Wei

**Affiliations:** Business School, Nanjing University, Nanjing, China

**Keywords:** CEO entrepreneurial orientation, middle management team innovativeness, social information processing theory, fsQCA, digital entrepreneurship era

## Abstract

Social information processing theory suggests that the chief executive officer’s entrepreneurial orientation (CEO EO) is an organisational signal that influences the members’ innovativeness. Middle management teams (MMTs) are expected to be more innovative as they connect senior managers with frontline managers in the dynamic competitive environment of the digital economy. How CEOs guide MMT innovations through EO becomes critical in the process of capturing opportunities and creating value. However, previous research has failed to adequately identify distinct CEO EO manifestations with organisational contexts configurations that influence MMTs innovation. Thus, based on differences in organisational contexts and MMTs’ cognition, this study thoroughly investigates how the vertical manifestation of CEO EO impacts the innovativeness of MMTs. We used fuzzy-set qualitative comparative analysis (fsQCA) on a sample of 117 organisations to determine which configurations of CEO EO vertical penetration within an organisation can stimulate MMT innovativeness. The study discovered four first-level configurations that support stimulating MMT innovativeness respectively when the CEO EO is fully or partially manifested, and without the CEO EO. Moreover, we found the internal reasons for MMTs’ information interpretation heterogeneity, which is critical for realising the coordination and unity of entrepreneurial cognition and behaviours. Finally, these findings’ theoretical and practical implications are discussed.

## Introduction

Chief executive officer entrepreneurial orientation (CEO EO) is an emerging topic in entrepreneurship research (Liu and Xi, 2021; Liu et al., 2021). Several studies have found that CEOs with high EO have a positive impact on their organisations (Keil et al., 2017). Although we are becoming more aware of the benefits of CEO EO, the literature on innovative, CEO personal outcomes brought about by CEO EO is significantly less developed. Furthermore, the role that CEO EO can play in the complex business environment and various organisational contexts is awaiting in-depth exploration of new research methods. Moving forward, it is critical to gain a better understanding of the innovative and CEO personal consequences of CEO EO (Liu and Xi, 2021; Liu et al., 2021).

Research on the role of the CEO EO within the organisation must be discussed in a specific context to be meaningful (Wales et al., 2020; Liu and Xi, 2021). Digital technology developing is currently the most important trend that businesses must deal with. Digital technology penetrates deep into the core of product and service operations. It fundamentally changes the nature of product and service innovation, making digitalisation an essential component of enterprise innovation processes (Yoo et al., 2012). While the digital economy brings innovation opportunities and value to enterprises, it also creates difficulties and challenges. Today’s enterprises are operating in a complex and fast-paced innovation environment due to the rapid rate of changes and uncertainties. Liu and Xi (2021) suggest that CEO EO can manifest CEOs’ entrepreneurial spirit and high commitment. CEO EO can achieve top-down penetration within the organisation and has an impact on other members’ innovative behaviour (Wales et al., 2011). Faced with the uncertainties and challenges of the digital economy era, how CEOs guide the cognition and behaviour of members through EO become critical in the process of capturing opportunities and creating value (Plsek, 2003).

An information-based and team-centric characteristic structure is the dominant trend for developing relationships between organisational members in the digital innovation era (Drucker, 1999). The uncertainty environment makes strategic decision-making and execution difficult, particularly for management teams (Plsek, 2003). Especially, middle management teams (MMTs) play a critical role in organisational management. The processes used by middle managers to obtain information quickly, while achieving full transmission and sharing within organisations, are critical in the digital entrepreneurship era. As the “horizontal information brokers and capability integrators” who connect senior managers with frontline managers, MMT innovativeness and management skills are increasingly being demanded (Tseng et al., 2019).

However, Plsek (2003) argues that MMTs are more likely to choose traditional management models, which emphasises the safety of adhering to standard operating procedures but produces a general lack of initiative for innovation in the changing environment. Furthermore, it is difficult for MMTs to form unified cognitive models because of the different department functions; this is not conducive to effective communication and information sharing among organisation members (Bartram, 2000). Exploring how to increase MMT innovativeness helps companies better deal with the challenges posed by the digital economy.

Chief executive officer entrepreneurial orientation is important to MMT innovativeness (Wales et al., 2020; Liu and Xi, 2021). According to social information processing theory, CEO EO is a significant source of organisational information (Lau and Liden, 2008) and affects MMT innovativeness (Wales et al., 2011). However, MMTs interpret CEO EO differently due to different situations within and outside organisations, as well as differences in individual perceptions (Wales et al., 2011). This cognitive difference affects how information is transmitted and shared within organisations, which impacts entrepreneurship outcomes differently (Kuratko et al., 2005). Therefore, this study explores the impact of CEO EO vertical penetration on MMT innovativeness in different settings. CEO EO facilitates information sharing and transmission within organisations and assists internal managers in dealing with the challenges brought about by the digital economy.

In sum, this study investigates the configurations that affect the relationship between CEO EO and MMT innovativeness in the digital entrepreneur era. The external environment is complex and dynamic; consequently, organisational structures become flattened to adapt to dynamic and competitive changes (Rajan and Wulf, 2006). Furthermore, performance pressure is exacerbated by complexity and changes environment. Thus, the dynamic competitive environment is the external situational condition in this study, and organisational structure and performance pressure are internal situational conditions. Then, we consider MMTs cognition: confidence in the organisation’s prospects and achievement orientation. Our research method, fuzzy-set qualitative comparative analysis (fsQCA), considers both configuration comparison and set theory, treats social phenomena as a complex combination of attributes, and investigates “multiple concurrent causalities” as a result of the set relationship (Ragin, 2000). Considering the external and internal environment, and personal factors involved in the research question, fsQCA is suitable.

The main contributions are as follows. First, based on the vertical penetration perspective of CEO EO, this study specifically investigates how CEO EO impacts MMT innovativeness, which contributes to the CEO EO research literature. Second, using the digital economy as a backdrop, this study investigates how to improve MMT innovativeness in response to the complexity environment, which has both theoretical and practical implications. Third, using social information processing theory, this study aims to unlock the CEO EO vertical penetration model’s black box in terms of MMT innovativeness, thereby giving a novel theoretical approach for EO research. Fourth, this study employs fsQCA to thoroughly examine the various configuration pathways that CEO EO has on MMT innovativeness. This is because fsQCA enables the evaluation of multiple concurrent causalities by identifying context-specific causal paths that lead to the same outcome. Thus, it is possible to acquire a deeper understanding of the internal process of CEO EO vertical penetration into different levels of organisations.

## Literature Review

### Social Information Processing Theory

The essence of enterprise digital innovation is using a combination of information, computing, communication, and connectivity technologies in the innovation process, as well as the resultant new products, improved production processes, changes in organisational models, and creation of innovation models (Nambisan, 2017). The digital entrepreneurial era has produced disruptive changes in the subject and elements of innovation, the innovation process, and the innovation platform. This is because digital technology enables organisations to start searching for rules and summarising knowledge from big data and then apply the knowledge and use it to accomplish specific goals and tasks. To deal with the impact of external uncertainties, the development of digital technology innovation companies increasingly relies on information provided by massive amounts of data (Haenlein and Kaplan, 2019).

According to social information processing theory, the process of cognition formation involves individuals processing information on external things (Salancik and Pfeffer, 1978). Bandura (1986) thinks that persons’ social attributes determine the interaction between humans and the environment. The interaction of individuals, external situations, and the organisational environment impacts personal cognition and behaviour. Furthermore, Gurbin (2015) suggests that the specific characteristics and environments of individuals significantly impact how an individual processes information; this influence runs through every stage of information processing. Thus, in a dynamic and complex environment, individuals rely on information provided by their social information environment to adjust their attitudes and behaviours (Salancik and Pfeffer, 1978). In the digital entrepreneurship era, when confronted with complicated digital information, organisational members typically demand the ability to quickly process data to realise their entrepreneurial consciousness and better seize market opportunities. Specifically, organisational members should receive, store, encode, convert, recycle, and transmit received information through a series of processing links to continuously improve their innovativeness (Wyer and Srull, 1986). In this process, members’ innovation attitudes and behaviours are influenced not only by their needs and goals but also by the surrounding environment. Moreover, when confronted with uncertainty and complexity in the digital economy era, individuals are more optimistic about obtaining social information regarding innovative attitudes and behaviours from their social environment.

Organisational models have changed in the digital economy, and teamwork has received increasing attention in the digital transformation of enterprises (Bouncken et al., 2021). Cognition is not limited to individuals and teams are also information processors (Hinsz et al., 1997). Teams form their cognition as a result of information sharing and integration among members. However, the cognition of individuals and teams differs significantly. Teamwork is a significant social context that influences individuals’ thinking, attitudes, and behaviours (Bhave et al., 2010). Therefore, social information processing theory researchers are currently focussing on how to coordinate innovativeness among teams and members (Rego et al., 2017). The process primarily consists of the following stages. First, based on the external context and development trend, organisational leaders deliver information to members who are compatible with the enterprise’s innovation strategy. Then, the information input. Individuals’ cognitive activities are triggered by external information. Specifically, individuals screen and enter data based on their prior experiences. Third, the cognitive subject pays attention to specific information selectively, because individuals typically cognise and process information through existing cognitive models. The fourth step is to re-encode, categorise, and interpret the information so that it can guide subsequent cognitive activities. Finally, the coordination and integration of various individual cognitions unifies individual and team cognition (Gurbin, 2015).

### Chief Executive Officer Entrepreneurial Orientation Vertical Penetration

Digital technology promotes organisational changes in the digital innovation age. Digital technology has produced changes in transaction processing, decision-making, office methods, and organisational forms. To remain competitive in the digital disruption era, firms should generate a durable competitive edge and prioritise the innovation capabilities, which are growing in tandem with technology advances (Salamzadeh et al., 2021). Furthermore, if enterprises want to achieve disruptive innovation and development, CEOs should coordinate the organisation’s internal resources as a whole and promote the integration of the organisation’s operation model with digital technology. As the primary decision-maker, the CEO’s cognition and execution ability are critical to realising a digital transformation and enhancing the enterprise’s competitive advantage (Liu and Xi, 2021). The key to digital transformations of companies is whether their CEOs can capture market changes and innovation opportunities, whether they are sensitive to innovation, and whether they can guide internal organisational members to form a cognition that matches digital innovation (Liu and Xi, 2021).

Chief executive officers develop their ongoing concern and willingness for innovation and entrepreneurship, also known as CEO EO, by receiving, filtering, interpreting, reacting, and processing environmental information (Hambrick and Mason, 1984). CEO EO reflects the CEO’s strong commitment to innovativeness, proactiveness, and risk-taking activities in the company’s innovation and entrepreneurship development processes (Keil et al., 2017). As an important source of internal information, CEO EO delivers market information to organisations (Rego et al., 2017; Liu and Xi, 2021). It has a significant impact not only on corporate innovation strategy decisions but also on others’ attitudes and behaviours.

Chief executive officer entrepreneurial orientation, a type of information, can penetrate vertically into different levels within an organisation and influence the innovation and entrepreneurship cognition, attitudes, and behaviours of organisational members (Rego et al., 2017; Wales et al., 2011, 2020; Liu and Xi, 2021). First, CEO EO is the core decision maker’s self-awareness, which influences the senior management team’s goals and directions, as well as the enterprise’s overall strategic decision-making for innovation (Keil et al., 2017). Employees need clear goals and tasks to activate their internal motivation for innovation. Second, the specific configuration of organisational elements influences CEO EO, and different organisational element configurations have different effects on employee innovative cognition and behaviour (Wales et al., 2020). Third, innovative CEOs set certain role expectations for their employees in the process of developing innovation and entrepreneurship. Furthermore, CEOs should use specific methods to align their entrepreneurial cognition with the organisation’s innovation and entrepreneurial cognition model. Specifically, CEO EO is shared and transmitted across organisational levels (Wales et al., 2011, 2020; Liu and Xi, 2021). It assists in unifying the CEO EO with the organisation’s cognitive model of innovation, ultimately motivating the innovative attitudes and behaviours of other members (Gurbin, 2015). Therefore, we think that CEO EO can vertically penetrate an organisation and is critical for enterprise digital innovation and entrepreneurship.

### Middle Management Team Innovativeness

According to social information processing theory, middle managers play an important role in an organisation’s input, processing, and sharing of information (Salancik and Pfeffer, 1978; Liu and Xi, 2021). The main task of an MMT as information flow facilitators is to ensure the effective transfer of information from top-level management to operating-level managers (Floyd and Lane, 2000). Middle managers accurately search, process, and integrate complex information, while interacting and coordinating to achieve effective communication and information sharing among organisational members. The knowledge spillover resulting from information transfer can serve as a catalyst for innovative activities (Ramadani et al., 2017). Moreover, it is important for deepening team members’ mutual coordination of values and cognition (Bhave et al., 2010; Ren and Guo, 2011).

Organisational forms become increasingly flat in digital innovation era. Relationships between organisational members are dominated by information-based, team-centred structures (Drucker, 1999); teams are now considered the norm for navigating complex environments (Salas et al., 2005). As intermediaries who connect the different levels of an organisation, how middle managers respond to changes in organisational development models, structure, and members’ relationships brought about by digital innovation is important for developing digital innovation in organisations (Hornsby et al., 2002). Furthermore, MMT innovativeness plays a significant role in identifying, improving, and guiding entrepreneurial opportunities, as well as in acquiring and allocating entrepreneurial resources (Ren and Guo, 2011). Therefore, it is difficult for the traditional MMT operating model to adapt to the changes and challenges enterprises face in the digital innovation era. Companies should stimulate the innovative thinking of MMTs if they are to fully realise their substantive role in the digital innovation process (Rego et al., 2017; Liu and Xi, 2021).

Some researchers have found that other members’ innovativeness, forms of information sharing, and methods of organisational element configuration influence the MMT innovativeness (Kuratko et al., 2005). Thus, the vertical penetration mode of CEO EO within an organisation impacts the innovation and behaviours of MMTs (Ren and Guo, 2011; Wales et al., 2011). Moreover, managers have different understandings of CEO EO due to differences working roles and functional scope (Wales, 2016; Liu and Xi, 2021). CEO can unify and guide the senior management team’s innovativeness directly (Liu and Xi, 2021). However, due to differences in their situations and characteristics, MMTs cognitive perspectives on CEO EO differ from those of senior managers, according to social information processing theory (Wales et al., 2011; Liu and Xi, 2021). Thus, researching how to encourage MMTs to positively interpret CEO EO plays a critical role in stimulating their innovativeness.

### Variables

According to social information processing theory, individuals’ or teams’ innovativeness, attitudes, and behaviours are influenced by the combination of external conditions, personal needs, and organisations’ internal environment (Salancik and Pfeffer, 1978). Therefore, in terms of contextual variables, this study thoroughly investigates the three areas of external contextual factors, internal organisational factors, and MMT cognition.

#### External Environment Variable: Dynamic Competitive Environment

Entrepreneurial orientation and innovativeness should be analysed in the context of the external environment, such as its dynamics (Engelen et al., 2014). External competition for businesses has grown stronger, and the market environment has become more diverse in the digital economy era (Rosenbusch et al., 2013). Firms often engage in entrepreneurial activities to ensure their success and survival in highly dynamic and competitive contexts (Dana et al., 2022). The dynamic environment of market competition significantly impacts corporate innovation and entrepreneurship. An enterprise’s dynamic competitiveness primarily includes two aspects: dynamics stresses the speed and instability of changes in the external environment (Barrales-Molina et al., 2010), while environmental competitiveness refers to the level of competition in a company’s external environment, including the number of competitors in the industry market and the market’s capacity (Mithas et al., 2013). As the dynamic competitive environment becomes more visible, organisations increase employees’ requirements to innovate and proactively recognise and capitalise on prospective market possibilities.

Based on social information processing theory, specific social information in the social environment is more likely to capture individual attention and consequently influence individual attitudes and behaviours (Bhave et al., 2010). Dynamically competitive markets have become the main trend in the digital economy, and enterprises should be innovative, proactive, and risk-taking when such an environment emerges (Rosenbusch et al., 2013). As a major source of information transmission in an organisation, CEOs are important in the process of identifying innovation prospects and making development plan decisions. Therefore, CEOs with an entrepreneurial orientation are more acutely aware of the dynamic and competitive changes in the market environment, allowing them to provide more accurate and comprehensive innovative market information to middle managers (Liu and Xi, 2021). Some scholars argue that when individuals perceive their surroundings as unstable, they rely more on the information provided by their surroundings to gain a sense of certainty and stability (Hogg, 2001). When a CEO is entrepreneurial, the organisational members’ innovativeness and entrepreneurial behaviours, as well as the entrepreneurial activities involved in value creation, are encouraged (Keil et al., 2017). Organisational members are more likely to constantly adjust their innovativeness and behaviours to match the information or signal their feelings of certainty and stability (Yang et al., 2018).

#### Internal Environment Variable: Organisation Structure

Research shows that the main factor that influences how CEO EO penetrates within an organisation is the organisational structure (Wales et al., 2011; Yoo et al., 2012; Wales, 2016). Organisational structures have shifted from vertical to flat, and an organisation’s internal governance mechanisms have become more democratic in the digital entrepreneurship era. This change impacts the degree of penetration of CEO EO, as well as how members of the organisation interpret and share information sources (CEO EO). Second, an autonomous organisational structure emphasises mutual trust, cooperation, and information sharing, which can ensure smooth communication, collaboration, and coordination between departments, as well as organic integration of various departments’ capabilities (Rhee et al., 2017). Thus, as information communicators, MMTs in an autonomous organisational structure can more effectively transmit CEO EO to other members, thereby realising individual cognition and team-wide cognition coordination (Wales et al., 2020). Third, an autonomous organisational structure transforms the flow of information and decision-making within an organisation from one-way to a flow that is widely spread throughout the organisation. This allows CEO EO to be fully perceived within the organisation, facilitating positive interactions among organisational members and encouraging enthusiasm and initiative for innovation (Wales, 2016). In addition, discovering and resolving problems is part of the innovation process when there is uncertainty and ambiguity. The essence of technological innovation is reducing uncertainty and ambiguity; however, achieving this goal requires information exchange and organisational resource support. An autonomous organisational structure promotes smoother information communication than a mechanical organisational structure and gives more autonomy to internal teams and individuals (Menon and Varadarajan, 1992), allowing for team innovation.

#### Performance Pressure

Managers and employees face a more complex working environment and increased corporate performance pressure to effectively adapt to the complexity and uncertainty environment (Neal and Hesketh, 1999). The work pressure caused by factors such as performance appraisals is referred to as performance pressure. The difference between the company’s expected performance and its potential performance creates performance pressure and causes individuals to be concerned about the company’s ability to meet its expected profit goal (Durham et al., 2000). On the one hand, performance pressure motivates managers and employees to work hard to obtain performance (Gardner, 2012); on the other hand, performance pressure forces them to improve performance to avoid the perceived negative consequences, emphasising that performance pressure is subjective (Mitchell et al., 2018).

Performance pressure has a dual effect within an organisation as it generates both positive and negative side effects (Mitchell et al., 2018). Moreover, previous research has demonstrated that performance pressure elicits both functional and dysfunctional behaviour (Eisenberger and Aselage, 2009). Therefore, we think that performance pressure causes dynamic changes in the attitudes and behaviours of organisational members. Based on social information processing theory, the team’s perception of performance pressure is transmitted within the organisation as a type of information. This information motivates team members to constantly assess the distance between themselves and the target task, which eventually leads to different innovative cognitions and behaviours (Kluger and DeNisi, 1996). When team members perceive performance pressure as threatening, they may develop negative emotions. However, positive cognitive behaviours, such as creativity and intrinsic interest, can be produced when team members view performance pressure as an intriguing challenge (Ganster and Rosen, 2013). Thus, we consider that regarding performance pressure as a causal variable to investigate its impact on MMT innovativeness is critical to organisational internal entrepreneurial activities in the digital innovation era.

#### Confidence in the Organisation’s Prospects

Confidence in the organisation’s prospects can be described as members’ positive evaluation of and belief in the organisation’s development (Liu and Xi, 2021). Based on information processing theory (Benjamini and Hochberg, 1995), members receive information from both inside and outside the organisation and judge its development prospects based on their cognition. First, when investigating the vertical penetration of CEO EO in organisations, CEOs that have an innovative and entrepreneurial orientation pass their ideas, plans, and actions to the organisation and then execute them (Covin and Slevin, 1989), with the ultimate goal of gaining more market share and excess profits (Monsen and Wayne Boss, 2009). Compared with grassroots employees, MMTs may learn information (CEO EO) earlier and more thoroughly. This is because MMTs are an important part of CEOs’ communication of innovative ideas and the implementation of innovative and entrepreneurial plans. If MMTs interpret CEO EO as positive information, they may put more effort into their work (Liu and Xi, 2021). Second, CEOs with an entrepreneurial orientation are more receptive to new ideas and suggestions for improving the implementation of innovation and entrepreneurship, as well as encouraging and supporting organisational members’ participation in innovative activities. These factors contribute to MMTs positive perceptions of an organisation’s prospects (Kellerman, 2008).

Middle management teams who are more confident in the organisation’s prospects are more willing to invest in team innovation. When CEOs vertically penetrate innovation, they send a message of seizing market profits and creating wealth (Monsen and Wayne Boss, 2009), which encourages MMTs to be optimistic about organisational innovation (Chaston and Sadler-Smith, 2012). The cognition of individuals influences their behaviours (Bandura, 1991). Thus, middle managers are more willing to improve team innovativeness when they have positive ideas about innovation and entrepreneurship.

#### Achievement Orientation

A person’s desire and psychological proclivity to overcome difficulties, achieve success, and pursue perfection are referred to as achievement needs; this is an important personal characteristic that encourages people to strive to realise worth (Murray, 1938). McClelland (1987) indicated that achievement motivation is an internal driving force that individuals acquire to attain success. McClelland’s research since the 1960s has shown that achievement needs are positively correlated with economic development and are closely related to innovation and entrepreneurship. In addition, McClelland and Burnham (2017) pointed out that achievement needs are important for the success of small business owners or managers. Thus, many researchers investigate achievement motivation as a key psychological characteristic of entrepreneurs or employees. This is because individuals with achievement orientation are more likely to be drawn to positions requiring innovation and entrepreneurship to meet their needs (MacKenzie and MacKenzie, 1995). Furthermore, some scholars think that innovative processes are real events that are influenced by complex social backgrounds and internal organisational contexts. Therefore, innovation research should incorporate the achievement orientation of entrepreneurs and employees into a complex situation for research (Weerawardena and Sullivan Mort, 2006). Finally, achievement-oriented motivation is regarded in this study as the psychological motivation that stimulates MMT innovativeness.

**FIGURE 1:**
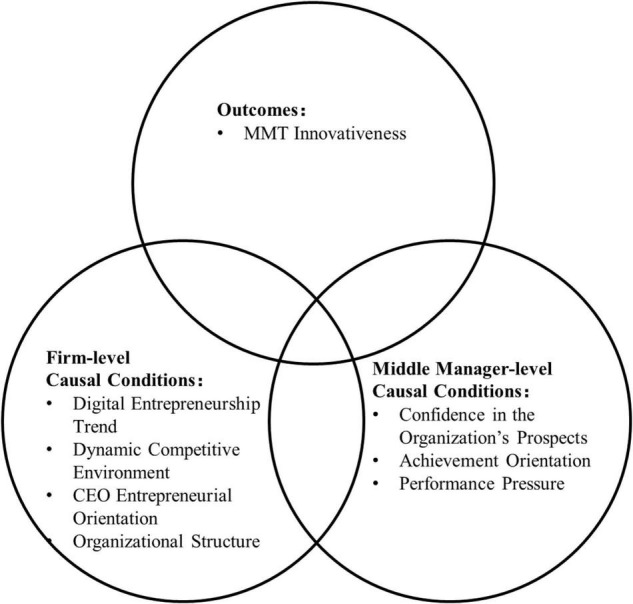
Conceptual model.

Based on the discussion, the conceptual model is presented in Figure 1.

## Methodology

### Sample

This study employs a multi-source research design to test how to shape the MMT innovativeness in the face of the complex and changing organisational environments, the CEO EO, and the personal characteristics of middle managers. Our sample enterprises from the four economic and technological development zones in China’s Yangtze River Delta Industrial Zone. Choosing the Yangtze River Delta Industrial Zone for two reasons: first, it is China’s largest comprehensive industrial base, with a developing high-tech industry; then, it has a high technological level and the most comprehensive structure in China. Thus, the area is rich in technological innovation resources.

Aside from the digital enterprise infrastructure construction investments in each province, the questionnaire used a 7-point Likert scale and was translated into Chinese and English using standard back-translation methods (Brislin, 1980). This questionnaire was intended to be completed by the company’s CEO and MMTs. Random sampling was used to interview 170 small and medium-sized enterprises (SMEs) based on the National Bureau of Statistics of China’s proprietary SME database from March to December 2019. During the data gathering procedure, we conducted additional tests and implemented a variety of checks to ensure that the questionnaire data accurately represent the measurement findings. A total of 128 SMEs participated in the survey; after 11 items were removed due to missing data, 117 cases were analysed. This questionnaire’s overall recovery rate was 68.88%. These companies’ average length of existence is 19 years. Among the 117 SMEs, 53 are manufacturing firms, 64 are high-tech firms, and 65 are service firms. There were 101 males among the 117 CEOs interviewed, with an average age of 46–50 years old and a tenure of 98 months, while 56 of the 117 mid-level managers interviewed were men, with a tenure of 55 months.

### Overview of Fuzzy-Set Qualitative Comparative Analysis

Fuzzy-set qualitative comparative analysis (fsQCA) is both a research method and a collection of analysis tools. It is a novel research method that combines the advantages of qualitative and quantitative methods (Ragin, 2000). On the one hand, fsQCA leverages the capabilities of qualitative research to elicit information directly from research subjects, hence minimising measurement error associated with survey research (Dana and Dana, 2005). On the other hand, fsQCA combines the benefits of quantitative analysis, resulting in reproducible study results (Douglas et al., 2020). In terms of methodology, it employs both configuration comparison and set theory (Ragin, 2000). fsQCA is beneficial for analysing asymmetric relationships between dependent and independent variables (Woodside, 2011, 2013). Consequently, fsQCA is engaged in the complexity of developing things and finding and identifying the causal path that leads to the same result in different situations to evaluate multiple concurrent causalities (Rihoux and Ragin, 2012). Scholars call for researchers to use of the fsQCA approach to a variety of micro and macro business concerns, such as innovation and entrepreneurship studies (Douglas et al., 2020). This is because fsQCA eliminates the assumption of independence between influencing factors, is compatible with cross-layer factor embedding (Greckhamer, 2011; Kraus et al., 2018), and does not require special cross-layer variable processing, making it particularly suitable for management research involving multi-layer variables (Morgan, 2010; Kraus et al., 2018). System theory suggests that because of imitation, coercion, and regulation, an organisation’s configuration tends to condense and cannot be infinitely varied (DiMaggio and Powell, 1983). Furthermore, the theory of social construction suggests that because people’s actions, motives, and behaviours are constantly repeated, some configurations will be selected and continually strengthened (Berger, 1967). Therefore, we employed fsQCA 3.0 for our analysis.

### Measures

The survey questionnaire contained an outcome variable regarding MMT innovativeness, as well as causal conditions from the external environment to the organisational characters and middle manager levels. We used a scale validated by previous research to assess MMT innovativeness with seven items (Atuahene-Gima et al., 2005; Chen et al., 2009). MMT innovativeness measurement primarily includes (1) the team’s innovative ideas and plans; (2) the team’s innovative work results; (3) the team’s innovative use of existing resources and information; and (4) the team’s current product or service improvement.

The external context was chosen based on the business trend and industry background. First, the business environment was chosen to be the mainstream trend of the digital economy. Based on the various China provinces panel data from China Statistical Yearbook, Shuaitao and Qiubi (2021) build a spatial measurement model to measure the provincial digital economy development index for China’s inter-provincial digital economy from four dimensions of digital foundation, application, innovation, and transformation. We use this indicator to determine the digital development trend in each province. Second, the dynamic competitive environment of the industry was chosen to evaluate the company’s industry background, which is measured with eight items (Zollo and Winter, 2002; Barrales-Molina et al., 2010).

In terms of firm-level organisational factors, CEO EO is used to capture corporate management’s innovation and entrepreneurial intentions, including innovativeness, proactivity, and risk-taking with nine items (Liu and Xi, 2021). The organisational structure is captures with seven items about the organisation’s freedom of information exchange, decision-making, and cooperation. Higher scores denote a more dynamic structure, whereas lower scores indicate a more mechanistic structure.

About manager-level causal conditions, four items are used to evaluate confidence in the organisation’s prospects (Liu and Xi, 2021). For example, one of the items is “I am confident that the company will develop better in the future.” Moreover, four items address achievement orientation motivation or a person’s desire and psychological proclivity to overcome difficulties, achieve success, and pursue perfection (Lang and Fries, 2006). Finally, four items from Charbonnier-Voirin and Roussel (2012) measure performance pressure. Scales are in the Supplementary Appendix A.

### Calibration

For configurational analysis, each variable should be calibrated for set membership (Ragin, 2013). Owing to the variation in the kurtosis and skewness each factor’s data set, this study employs percentages to directly establish the qualitative anchor point (Morgan, 2010). As part of this investigation, percentiles were utilised to calibrate the data. A threshold above 95% indicates that observations are “fully in” the set membership; a crossover point of 50% indicates that observations are “neither in nor out,” and a threshold below 5% indicates that observations are “fully out” of the set membership. Following recommended practices, we recalibrated each set with an exact membership score of crossover point, by introducing a tiny constant (0.001). Details in Table 1.

**TABLE 1:** Sets, calibrations, and descriptive statistics.

Sets	Fuzzy-set calibrations			Descriptive statistics					
	Full in	Crossover	Full out	Mean	SD	Min	Max	*N* cases	Missing
DCE	7	4.667	2.667	4.789	1.141	2.111	7	117	0
DEL	18.530	18.448	18.365	0.952	0.010	0.95	1	117	0
OS	6.857	4.714	2.571	4.779	1.213	1.429	7	117	0
Inn	7	6	3.6	5.707	1.128	2	7	117	0
Pro	7	6	4	5.934	0.999	2.333	7	117	0
RT	7	5.667	4	5.658	1.037	2.333	7	117	0
COP	7	6.125	4.463	6.033	0.996	0	7	117	0
AO	7	6	4	5.884	1.077	2	7	117	0
PP	6.525	5	2.250	4.788	1.339	0	7	117	0
TI	7	5.429	3.7	5.337	0.993	0	7	117	0

*DCE, Dynamic Competitive Environment; DEL, Digital Economy Level; OS, Organisational Structure; Inn, Innovativeness; Pro, Proactiveness; RT, Risk-taking; COP, Confidence in the Organisation’s Prospects; AO, Achievement Orientation; PP, Performance Pressure; TI, Middle Management Team Innovativeness.*

## Results

The fsQCA method includes two critical steps: a necessity test and a sufficiency test. These two steps determine the configuration of the necessary and sufficient conditions to promote the result in the presence of causal complexity.

### Analysis of Necessary Conditions

Whether each variable is a necessary condition for the outcome variable must be checked before constructing a sufficiency analysis. Conditions that should exist for results to occur are referred to as “necessary conditions.” As a standard fsQCA practice, fuzzy set analysis is performed on the necessary conditions, with a consistency benchmark of 0.90. Based on the necessary condition analysis in Table 2, the province digital economy level where the company located in is a necessary condition for MMT innovativeness.

**TABLE 2:** Analysis of necessary conditions for middle management team innovativeness in fuzzy-set qualitative comparative analysis.

Outcomes: MMT innovativeness		
Sets of conditions	Consistency	Coverage
DCE	0.676	0.654
∼DCE	0.676	0.689
DEL	0.999	0.521
∼DEL	0.094	0.975
OS	0.698	0.672
∼OS	0.653	0.669
Inn	0.717	0.698
∼Inn	0.604	0.611
Pro	0.729	0.652
∼Pro	0.576	0.642
RT	0.703	0.658
∼RT	0.610	0.644
COP	0.824	0.727
∼COP	0.507	0.574
AO	0.703	0.636
∼AO	0.578	0.636
PP	0.684	0.648
∼PP	0.645	0.671

*DCE, Dynamic Competitive Environment; DEL, Digital Economy Level; OS, Organisational Structure; Inn, Innovativeness; Pro, Proactiveness; RT, Risk-taking; COP, Confidence in the Organisation’s Prospects; AO, Achievement Orientation; PP, Performance Pressure. ∼ means the absence of. For example: ∼ Organisational Structure, absence of high OS.*

### Sufficiency Conditions Analysis

**FIGURE 2:**
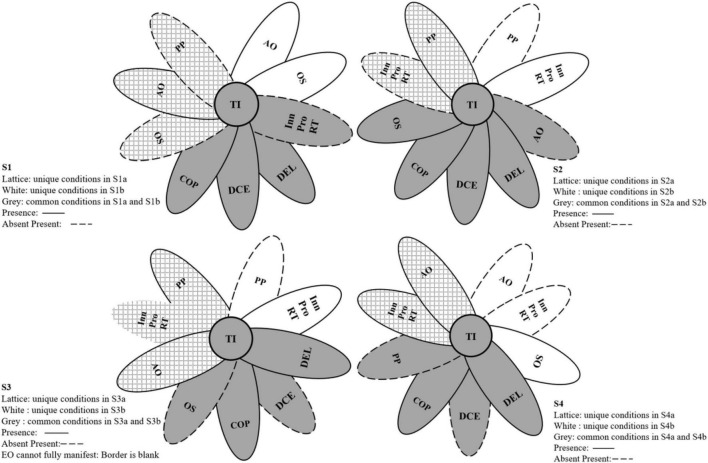
Configurations for high middle management team innovativeness (fuzzy-set qualitative comparative analysis). CEO EO, CEO Entrepreneurship Orientation (including Innovativeness, Proactiveness, and Risk-taking); OS, Organisational Structure; COP, Confidence in the Organisation’s Prospects; AO, Achievement Orientation; PP, Performance Pressure; TI, Middle Management Team Innovativeness.

The outcome of the adequacy test on the innovation stimulation of MMTs and fsQCA standard notation were used to report this investigation’s findings (Fiss, 2011). Table 3 shows that there are four first-level configurations and two second-level configurations in each group. Figure 2 shows that an ellipse with a black border indicates a condition is present, an ellipse with a dashed border indicates that the condition is absent, and no border indicates that EO cannot fully manifest (in S3a). The ellipse is not displayed if the condition is unrelated to the configuration. Grey represents the common conditions in second-level configurations, lattice marks alone represent Solution Xa (SXa), and white only represents Solution Xb (SXb). The raw consistency benchmark is set to greater than or equal to 0.8, and the inconsistency ratio reduction (PRI) is set to greater than or equal to 0.70, based on fsQCA operational requirements (Greckhamer et al., 2018). There are two sets of results, including configurations for high MMT innovativeness and for the absence of MMT innovativeness.

**TABLE 3:** Configurations for high middle management team innovativeness (fuzzy-set qualitative comparative analysis).

Configuration	Solutions							
	S1a	S1b	S2a	S2b	S3a	S3b	S4a	S4b
DCE	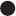	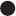	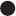	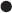	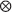	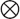	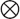	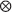
DEL	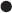	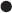	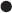	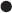	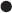	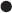	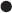	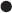
OS	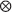	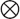	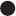	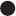	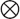	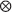		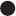
Inn	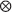	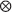	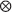	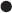	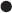	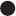	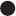	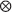
Pro	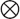	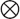	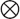	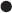	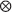	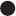	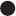	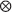
RT	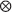	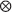	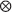	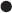	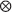	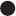	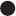	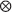
COP	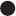	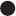	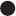	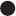	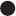	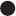	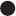	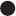
AO		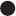	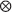	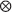	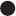		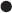	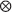
PP	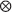		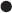	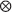	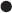	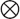	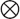	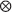
Raw coverage	0.248	0.231	0.236	0.221	0.181	0.283	0.317	0.212
Unique Coverage	0.007	0.009	0.031	0.016	0.012	0.006	0.050	0.018
Consistency	0.957	0.947	0.926	0.972	0.966	0.963	0.915	0.952
Solution Coverage: 0.536								
Solution Consistency: 0.906								

*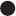, presence core conditions; 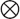, absence core conditions; ●, present contributing conditions; 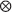, absence contributing conditions; blank, do not care; DCE, Dynamic Competitive Environment; DEL, Digital Economy Level; OS, Organisational Structure; Inn, Innovativeness; Pro, Proactiveness; RT, Risk-taking; COP, Confidence in the Organisation’s Prospects; AO, Achievement Orientation; PP, Performance Pressure.*

#### Configurations for High Middle Management Team Innovation

The solution coverage and consistency of MMT innovativeness were 0.536 and 0.906, respectively. Solution 1 (including S1a and S1b) and Solution 2 (including S2a and S2b) are configurations that stimulate MMT innovation in a dynamic competitive environment, whereas S3 (including S3a and S3b) and S4 (including S4a and S4b) provide pathways for encouraging MMT innovation in SMEs in a non-dynamic competitive environment. Moreover, because the level of the digital economy is a necessary condition, it exists in all configurations.

The dynamic competitive environment is an important causal factor in MMT innovativeness, as demonstrated by solutions 1 (S1) and 2 (S2). When companies with mechanised organisational structures operate in a dynamic competitive environment, the CEO’s innovation strategy is influenced by external competitive pressure (Covin and Slevin, 1989). A mechanised organisational structure is not conducive to the vertical penetration of CEO EO within an organisation and impacts the full display of CEO EO as an information source within the organisation (Wales et al., 2011). On the one hand, middle managers may be unable to perceive and transmit superior innovation strategy information due to a lack of accurate information sources (CEO EO) (Wales, 2016). On the other hand, a rigid organisational structure limits middle managers’ rights, which affects communication and information sharing between middle managers and subordinate employees (Rhee et al., 2017), which makes it difficult for middle managers to input, process, and share information. Hinder efficient information transmission and sharing put middle managers at disadvantage when it comes to achieving team member coordination and unity of cognition. On the one hand, CEOs can stimulate MMT innovativeness by reducing middle management’s perception of performance pressure as a threat (Mitchell et al., 2018) and cultivating middle management’s confidence in the organisation’s development (S1a). On the other hand, CEOs can foster innovativeness by encouraging the confidence in the organisation’s development and the achievement-oriented motivation of team members (S1b). The coverage of S1a and S1b is 0.248 and 0.231, respectively; and S1a and S1b’s consistency is 0.957 and 0.947, respectively.

When companies have autonomous organisational structures in a dynamic competitive environment, external competitive pressure affects the CEO’s entrepreneurship cognition and behaviour (Covin and Slevin, 1988), but an autonomous organisational structure may facilitate the vertical penetration of CEO EO (Wales et al., 2011). The CEO is an important source of information within an organisation, owing to the trend of digital innovation. If the CEO lacks entrepreneurial orientation, MMTs lack information sources about the organisation’s internal innovation and entrepreneurship. However, autonomous organisations have established that MMTs have decision-making power, which enables them to realise information sharing and communication among members (Rhee et al., 2017). Thus, MMT enthusiasm for innovation is critical to the team’s innovativeness model and atmosphere. Chief executive officers can clarify mission goals by improving MMT challenging perceptions of performance pressure, while also cultivating their confidence in the organisation’s development prospects, which is critical for stimulating their innovativeness (S2a, coverage is 0.236, consistency is 0.926). Furthermore, if there is the vertical pervasiveness of CEO EO, it will help MMTs obtain clear digital innovation strategy information, achieve top-down information sharing and transmission, and effectively supervise and control employee behaviour in subordinate departments (Liu and Xi, 2021). Thus, if the MMTs are full of confidence and enthusiasm for the organisation’s development prospects, it will stimulate MMT innovativeness (S2b, coverage is 0.221, consistency is 0.972).

Solutions 3 and 4 are strategies for promoting MMT innovation when the dynamic competitive environment has no significant influence. When the external environment is non-dynamic and non-competitive and the organisational structure lacks autonomy, the competitive pressure of the external environment has little impact on CEO entrepreneurship cognition and behaviour (Covin and Slevin, 1988). CEOs’ cognition and decision-making regarding the digital innovation trend are important in mechanical organisational structures. MMTs may not perceive superior innovation strategy information if CEO EO does not manifest sufficient vertical penetration in mechanical organisational structures. It is necessary to improve middle managers’ perceptions of performance pressure while also cultivating their confidence in the organisation’s prospects and stimulating MMT achievement orientation to boost their innovativeness (S3a). However, if CEO EO is fully manifested and achieves vertical penetration within an organisation (Wales et al., 2011), middle managers will be able to obtain clear goal-oriented directions regarding digital innovation and entrepreneurship. In the solution, if the MMTs are optimistic and confident about the organisation’s development prospects, it will stimulate MMT innovativeness (S3b). S3a (0.966) and S3b (0.963) have higher consistency than the accepted threshold of 0.80. The coverage of S3a and S3b is 0.181 and 0.283, respectively.

External environmental pressure has less impact on the CEO’s entrepreneurship when the external environment is non-dynamic and non-competitive. Regardless of organisational structure, CEOs should reduce the threat MMTs perceive from performance pressure to achieve innovativeness. Furthermore, if CEO EO can penetrate vertically within an organisation and encourage the use of digital equipment for innovative behaviours, MMTs will have high achievement orientation and be confident in the organisation’s development, which is essential for inspiring team innovativeness (S4a coverage is 0.317, consistency is 0.915). However, although CEO EO cannot be fully manifested in an organisation with an autonomic organisational structure, middle managers have some autonomy and participation rights in developing innovation and entrepreneurship, and internal organisational information can be shared and innovated. Thus, MMTs must capture information on the trends in digital innovation and entrepreneurship by themselves; when MMTs have full confidence in the organisation’s development prospects, it helps stimulate the team’s innovativeness (S4b coverage is 0.212, consistency is 0.952).

#### Configurations for the Absence of Middle Management Team Innovativeness

According to the asymmetry principle in fsQCA, we consider that a configuration that promotes teams’ innovativeness may differ from configurations that are absent of MMT innovation. Thus, we conducted a separate analysis of pathways where the MMT innovation is absent (Table 4). If the results are accurate, they mean that the pathways that promote innovation in MMTs are distinct from the cause of the absence of innovation in MMTs (e.g., Du and Kim, 2021). The results highlight the importance of confidence in organisational development. Regardless of the situation, if middle leaders lack confidence in the organisation’s development prospects, it may lead to insufficient or lack of innovativeness in a team.

**TABLE 4:** Configurations for the absence of middle management team innovativeness (fuzzy-set qualitative comparative analysis).

Configuration	Solutions				
	AS1a	AS1b	AS1c	AS2a	AS2b
DCE	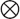	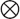	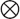	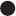	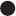
DEL	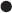	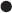	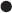	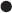	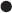
OS	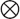	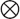	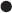	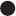	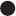
Inn	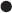	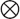	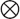	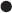	
Pro	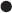	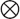	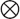	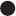	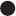
RT	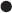	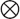	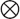	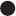	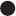
COP	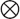	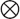	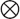	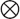	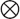
AO	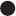	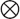		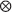	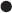
PP	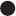		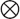	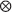	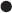
Raw coverage	0.192	0.263	0.244	0.273	0.251
Unique Coverage	0.017	0.056	0.015	0.039	0.030
Consistency	0.961	0.946	0.968	0.973	0.965
Solution Coverage: 0.478					
Solution Consistency: 0.945					

*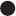, presence core conditions; 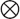, absence core conditions; ●, present contributing conditions; 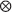, absence contributing conditions; blank, do not care; DCE, Dynamic Competitive Environment; DEL, Digital Economy Level; OS, Organisational Structure; Inn, Innovativeness; Pro, Proactiveness; RT, Risk-taking; COP, Confidence in the Organisation’s Prospects; AO, Achievement Orientation; PP, Performance Pressure.*

When the dynamic competitive environment has no significant impact on companies and CEO EO can be manifested in a mechanical organisation, MMTs are under performance pressure and achievement orientation but lack confidence in the company’s development, resulting in low or no MMT innovation (AS1a). The absence of MMT innovation in 1b (AS1b) and 1c (AS1c) demonstrate that, regardless of organisational structure, if there is no CEO EO and the necessary personal characteristics of MMTs are lacking, the MMT innovativeness cannot be stimulated.

In a dynamic competitive environment, the absence of MMT innovation in 2a (AS2a) demonstrates that when CEO EO is fully manifested, MMTs lack of confidence in the organisation’s prospects, lack of achievement orientation, and lack of performance pressure causes their innovation to vanish. Furthermore, even in the presence of performance pressure and achievement orientation, the solution in the absence of MMT innovation in 2b (AS2b) indicates that when CEO EO cannot fully manifest and incorporate vertical penetration, MMTs lack of confidence in the organisation’s prospects causes MMT innovativeness to disappear.

### Robustness Checks

The results of stimulating MMT innovativeness were subjected to a robustness test. The study was repeated after modifying the calibration points in all cases to 10 (full out), 50 (crossover), and 90 (full in) percentage points using the direct calibration method. The outcomes were similar. Details are in Supplementary Appendix B.

## Discussion, Implications, Limitations, and Future Research

### Discussion

**FIGURE 3:**
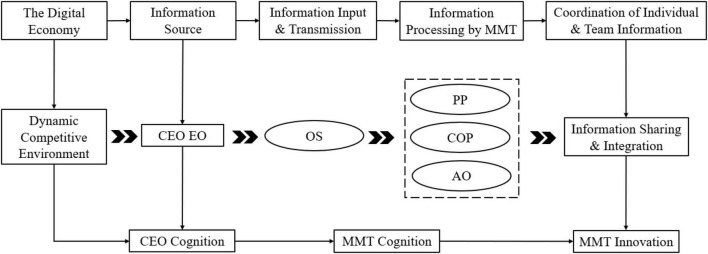
Chief executive officer entrepreneurial orientation vertical penetration and middle management team innovativeness. CEO EO, CEO Entrepreneurship Orientation; OS, Organisational Structure; COP, Confidence in the Organisation’s Prospects; AO, Achievement Orientation; PP, Performance Pressure.

The following conclusions were drawn based on the fsQCA configuration analysis. First, the complexity and variability of the market environment has a profound impact on the innovative cognition and behaviours of organisational members in the digital economy era (Wang et al., 2021). Second, with the growing diversity and complexity of external information, organisational members’ work becomes more difficult, and the importance of teamwork among members has begun to be emphasised. When confronted with complex digital information, middle managers should strengthen teamwork and cultivate the unity of innovativeness among team members. Third, according to social information processing theory, CEOs act as an important information channel, and CEO EO has a top-down impact on members’ innovativeness. This mode of influence is essentially CEO EO vertical penetration within the organisation. In addition, to achieve consistency in individual and team cognition, MMTs input, process, and share the information received (CEO EO) among team members based on the specific characteristics of the organisation’s internal situation and their perceptions. In turn, CEO EO can stimulate MMT innovativeness. Figure 3 shows the relationship between CEO EO vertical penetration and MMT innovativeness.

### Theoretical Implications

First, the external environment’s complexity and variability significantly impact enterprises’ internal innovation activities. This study investigates the relationship between CEO EO and MMT innovativeness and provides a new perspective for research on internal entrepreneurship during the digital innovation period. Second, based on social information processing theory, this study proposes that CEO EO, as organisations’ internal information source, has a significant impact on organisational members’ innovativeness (Wales et al., 2020; Liu and Xi, 2021). Moreover, this study provides the first in-depth interpretation of the internal mechanism of the top-down influence of CEO EO on MMT innovativeness and opens the black box of the vertical penetration of CEO EO to the recognition of MMT innovativeness. This adds to research on the role of EO penetration at various enterprise levels. Third, MMTs are important for organisational communication and information-sharing (Liu and Xi, 2021). This study investigates the internal reasons for MMTs’ information interpretation heterogeneity, which is critical for realising the coordination and unity of entrepreneurial cognition and behaviours. Finally, unlike previous single and fragmented research findings (Covin and Lumpkin, 2011), using the causal conditions in a dynamically competitive market, organisational structure, and middle manager cognition, this study is the first to employ fsQCA to investigate the path configuration of the innovativeness relationship between CEO EO and MMTs, which provides a better understanding of the internal mechanism of CEO EO vertical penetration. Furthermore, fsQCA uses Boolean algebra laws to collect the factors that drive the results and truly explain how CEO EO vertical penetration within an organisation impacts MMT innovation in a complex real-world environment. This contributes to the derivation and theoretical innovation of information processing theory and CEO EO in real business.

### Managerial Implications

This study’s findings have significant managerial implications. First, given the importance of CEOs’ continuous attention to entrepreneurial activities, companies should include the characteristics of CEO EO in the scope of investigation when selecting CEOs. Second, the results indicate that teamwork is an important way for organisations to respond to the complex and dynamic environment in the age of digital innovation. Therefore, it is critical to understand the unity of information sharing, cognition, and behavioural patterns among team members. Third, considering the importance of the external market environment for a company’s development, corporate decision-makers such as CEOs should pay attention to the external environment and take steps to address the challenges it poses. Finally, this study reveals that MMTs with different cognitive models have different innovativeness based on their perceived performance pressure, degree of organisational development confidence, and achievement-oriented behaviours. Thus, regarding entrepreneurship, organisations should pay more attention to how to motivate MMTs innovativeness.

### Limitations and Future Research

First, we encourage scholars to research the content of an organisation’s internal entrepreneurial activities. With the onset of the digital innovation era, organisational innovation efforts face new challenges. Whether in the organisational innovation development process or form, or in the innovativeness of organisational members, there is greater complexity and uncertainty. Thus, conducting in-depth study on a company’s internal entrepreneurial activities can help it adapt to the difficulties posed by digital innovation. Second, this study focuses on the vertical penetration of CEO EO within organisations; the findings show that CEO EO can achieve vertical, horizontal, and cross-time penetration in organisations (Wales et al., 2011). Therefore, we hope that scholars can research the role of CEO EO in the entrepreneurial process within organisations from a variety of perspectives. Furthermore, investigating how to fully manifest CEO EO within organisations aids in expanding the theoretical research framework of EO in the digital innovation era. Third, as CEOs’ cognitive model, CEO EO can influence the cognitive models and behaviours of employees at all levels of organisations (Liu and Xi, 2021). However, this study is the first to look at the impact of CEO EO on MMT innovativeness only. Some studies show that CEO EO influences the cognition and behaviour patterns of operations managers and front-line employees (Wales et al., 2011). Scholars can dig deeper into CEO EO vertical penetration and discuss it in more depth. Finally, this study employs fsQCA to examine how the vertical penetration of CEO EO promotes MMTs innovativeness. This study, however, is based on a static state, whereas dynamic studies that incorporate the time dimension represent another fsQCA trend. Therefore, scholars can attempt to investigate the vertical penetration of dynamic CEO EO within an organisation during the life cycle of different organisations and how it impacts the members’ innovativeness.

## Data Availability Statement

The raw data supporting the conclusions of this article will be made available by the authors, without undue reservation.

## Author Contributions

XZ wrote the manuscript and understood data processing. YL built the study structure framework, guided study writing, and gave theoretical guidance. XG provided scientific research funding support. DW provided research data. All authors contributed to the article and approved the submitted version.

## Conflict of Interest

The authors declare that the research was conducted in the absence of any commercial or financial relationships that could be construed as a potential conflict of interest.

## Publisher’s Note

All claims expressed in this article are solely those of the authors and do not necessarily represent those of their affiliated organizations, or those of the publisher, the editors and the reviewers. Any product that may be evaluated in this article, or claim that may be made by its manufacturer, is not guaranteed or endorsed by the publisher.
