# Supplementary material for: The Digital Entrepreneurship Era: How to Motivate Innovativeness in Middle Management Teams? The Vertical Organisational Pervasiveness of Chief Executive Officer Entrepreneurial Orientation
Source: Front Psychol. 2022 Mar 30;13:775558. doi: 10.3389/fpsyg.2022.775558 (PMC9009334; doi:10.3389/fpsyg.2022.775558)
Supplement: Supplementary file 1 [file Table_1.DOCX]

**Appendix A.** Scales

| **Variable** | **Item** | | | **Source** |
| --- | --- | --- | --- | --- |
| Provincial Digital Economy Development Index | Digital foundation | Mobile  Infrastructure | Mobile phone exchange capacity. | (Shuaitao & Qiubi, 2021) |
|  |  |  | Mobile phone penetration rate. |  |
|  |  | Fixed Infrastructure | Internet broadband access port. |  |
|  |  |  | Number of domains. |  |
|  |  |  | Number of sites. |  |
|  |  |  | Long distance optical cable. |  |
|  |  |  | Cable TV transmission trunk network total length. |  |
|  | Digital application | Digital Media | Number of digital TV users. |  |
|  |  |  | Proportion of the actual number of cable radio and TV users. |  |
|  |  | Enterprise Application | Number of corporate websites. |  |
|  |  |  | Number of websites owned by every 100 companies. |  |
|  |  |  | Proportion of the number of enterprises engaged in e-commerce transactions. |  |
|  |  | E-commerce Service | Total online retail sales/total retail sales of consumer goods. |  |
|  | Digital Entrepreneurship | Innovation Investment | Full-time equivalent of R&D personnel. |  |
|  |  |  | R&D funding. |  |
|  |  | Innovation  Output | Invention patent application/company patent application. |  |
|  |  |  | Technology market turnover. |  |
|  | Digital transformation | E-commerce Development and Reform | Software business revenue/GDP. |  |
|  |  |  | E-commerce sales/GDP. |  |
|  |  |  | E-commerce purchases/GDP. |  |
|  |  | New Product Benefit Reform | Number of new product development projects. |  |
|  |  |  | New product development expenditure. |  |
|  |  |  | New product sales revenue |  |
| Dynamic Competitive Environment | DCE1 | | Customer preferences shift quickly over time. | (Barrales-Molina et al., 2010; Zollo & Winter, 2002) |
|  | DCE2 | | Market demand and consumer preferences have always been volatile. |  |
|  | DCE3 | | Domestic and foreign competitors of the company have taken highly unpredictable actions Competition changes quickly. |  |
|  | DCE4 | | It's difficult to predict where this technology will go in the next five years. |  |
|  | DCE5 | | The degree of change in the external environment is massive or significant. |  |
|  | DCE6 | | Customers in the market frequently make new product demands (services) |  |
|  | DCE7 | | The external environment is always changing. |  |
|  | DCE8 | | The number and variety of products (services) available on the market are constantly evolving. |  |
| CEO EO | Inn1 | | Our CEO emphasizes innovative solutions to problems. | (Liu & Xi, 2021) |
|  | Inn2 | | Our CEO pays attention to innovation and update of products and/or services. |  |
|  | Inn3 | | Our CEO emphasizes investments in new technologies, methods, and procedures. |  |
|  | Pro1 | | In the face of market competition, our CEO emphasizes being proactive rather than reactive. |  |
|  | Pro2 | | Our CEO emphasizes the proactive introduction of new products, services, technologies, and business models. |  |
|  | Pro3 | | Our CEO emphasizes active identification and exploitation of new business opportunities by taking advantage of them. |  |
|  | RK1 | | Our CEO is willing to take higher risks in pursuit of higher returns. |  |
|  | RK2 | | Our CEO has a high tolerance for future uncertainty and is not afraid to change. |  |
|  | RK3 | | Our CEO is willing to take the risk of failure when setting up new projects or ventures. |  |
| Organisational  Structure | OS1 | | Important information within the company is transmitted very freely with unrestricted communication channels. | (Khandwalla, 1977; Rhee et al., 2017) |
|  | OS2 | | Managers work in a variety of ways, some of which are very formal and some of which are very informal. |  |
|  | OS3 | | Even if you ignore line managers for the time being, rely on expert decision-making. |  |
|  | OS4 | | Special emphasis is placed on adapting to environmental changes without relying too heavily on past practices. |  |
|  | OS5 | | Even if formal procedures are ignored, a strong emphasis is placed on getting things done. |  |
|  | OS6 | | Organizational control is loose/informal, with a focus on informal relationships and cooperation standards. |  |
| Confidence in the Organization’s Prospects | COP1 | | I am confident that the company will develop better in the future. | (Liu & Xi, 2021) |
|  | COP2 | | I believe our top management team, including the CEO, has the ability to lead our company forward. |  |
|  | COP3 | | I have confidence in my company’s strategy and business model. |  |
|  | COP4 | | I am confident that our company can cope with the competition in the industry. |  |
| Performance Pressure | PP1 | | My performance at work is very stressful. | (Charbonnier-Voirin & Roussel, 2012) |
|  | PP2 | | I feel the tremendous pressure from work. |  |
|  | PP3 | | I may be fired due to poor job performance. |  |
|  | PP4 | | My team and supervisor focus on work results. |  |
| MMT Innovativeness | MT1 | | The department's or team's ideas and solutions are far superior to those proposed by individuals. | (Atuahene-Gima et al., 2005; Chen et al., 2009) |
|  | MT2 | | The work of the department or team is innovative. |  |
|  | MT3 | | The department or team has produced a large number of unique and useful work results. |  |
|  | MT4 | | The department's or team's output demonstrates that we can make creative use of available resources and information. |  |
|  | MT5 | | The department or team has come up with a number of innovative solutions to the problem. |  |
|  | MT6 | | Members of a department or team can improve existing products, procedures, or create new ones. |  |
|  | MT7 | | Members of a department or team put in place appropriate plans and procedures to carry out new ideas. |  |

**Appendix B.** Robustness checks of configurations for team innovativeness outcome conditions (fsQCA).

| Configuration | Solutions |  |  |  |  |  |  |  |  |
| --- | --- | --- | --- | --- | --- | --- | --- | --- | --- |
|  | R1a | R1b | R2 | R3a | R3b | R4a | R4b | R4c |  |
| DCE | ● | ● | ● | ⊗ | ⊗ | ⊗ | ⊗ | ⊗ |  |
| DEL | ● | ● | ● | ● | ● | ● | ● | ● |  |
| OS | ⊗ | ⊗ | ● | ⊗ | ⊗ | ● |  | ● |  |
| Inn | ⊗ | ⊗ | ● | ● | ● | ⊗ | ● | ● |  |
| Pro | ⊗ | ⊗ | ● | ⊗ | ● | ⊗ | ● | ● |  |
| RT | ⊗ | ⊗ | ● | ⊗ | ● | ⊗ | ● | ⊗ |  |
| COP | ● | ● | ● | ● | ● | ● | ● | ● |  |
| HAM |  | ● | ⊗ | ● |  |  | ⊗ | ● |  |
| PP | ⊗ |  | ⊗ | ● | ⊗ |  | ⊗ | ● |  |
| Raw coverage | 0.195 | 0.189 | 0.152 | 0.139 | 0.218 | 0.164 | 0.258 | 0.143 |  |
| Unique Coverage | 0.009 | 0.119 | 0.022 | 0.113 | 0.008 | 0.030 | 0.0437 | 0.018 |  |
| Consistency | 0.934 | 0.922 | 0.957 | 0.946 | 0.942 | 0.948 | 0.885 | 0.925 |  |
| Solution Coverage: 0.464 | | | | | | | | | |
| Solution Consistency: 0.884 | | | | | | | | | |

Note: ●= condition present; ⊗ = condition absent; blank = condition either present or absent;

DCE=Dynamic Competitive Environment; DEL=Digital Economy Level; OS=Organizational Structure; Inn=Innovativeness; Pro=Proactiveness; RT=Risk-taking; COP=Confidence in the Organization’s Prospects; HAM=High Achievement Motivation; PP=Performance Pressure.
